# Long-Term Beta-Blocker Therapy in Patients With Stable Coronary Artery Disease After Percutaneous Coronary Intervention

**DOI:** 10.3389/fcvm.2022.878003

**Published:** 2022-05-17

**Authors:** Seung-Jun Lee, Dong-Woo Choi, Choongki Kim, Yongsung Suh, Sung-Jin Hong, Chul-Min Ahn, Jung-Sun Kim, Byeong-Keuk Kim, Young-Guk Ko, Donghoon Choi, Eun-Cheol Park, Yangsoo Jang, Chung-Mo Nam, Myeong-Ki Hong

**Affiliations:** ^1^Severance Cardiovascular Hospital, Yonsei University College of Medicine, Seoul, South Korea; ^2^Department of Preventive Medicine, Yonsei University College of Medicine, Seoul, South Korea; ^3^Cancer Big Data Center, National Cancer Control Institute, National Cancer Center, Goyang, South Korea; ^4^Seoul Hospital, Ewha Womans University College of Medicine, Seoul, South Korea; ^5^Myongji Hospital, Hanyang University College of Medicine, Goyang, South Korea; ^6^CHA Bundang Medical Center, CHA University College of Medicine, Seongnam, South Korea

**Keywords:** percutaneous coronary intervention, coronary artery disease, beta-blocker, drug-eluting stents, treatment outcome

## Abstract

**Background:**

It is unclear whether beta-blocker treatment is advantageous in patients with stable coronary artery disease (CAD) who underwent percutaneous coronary intervention (PCI). We evaluated the clinical impact of long-term beta-blocker maintenance in patients with stable CAD after PCI with drug-eluting stent (DES).

**Methods:**

From a nationwide cohort database, we identified the stable CAD patients without current or prior history of myocardial infarction or heart failure who underwent DES implantation. An intention-to-treat principle was used to analyze the impact of beta-blocker treatment on long-term outcomes of major adverse cardiovascular events (MACE) composed of cardiovascular death, myocardial infarction, and hospitalization with heart failure.

**Results:**

After stabilized inverse probability of treatment weighting, a total of 78,380 patients with stable CAD was enrolled; 45,746 patients with and 32,634 without beta-blocker treatment. At 5 years after PCI with a 6-month quarantine period, the adjusted incidence of MACE was significantly higher in patients treated with beta-blockers [10.0 vs. 9.1%; hazard ratio (HR) 1.11, 95% CI 1.06–1.16, *p* < 0.001] in an intention-to-treat analysis. There was no significant difference in all-cause death between patients treated with and without beta-blockers (8.1 vs. 8.2%; HR 0.99, 95% CI 0.94–1.04, *p* = 0.62). Statistical analysis with a time-varying Cox regression and rank-preserving structure failure time model revealed similar results to the intention-to-treat analysis.

**Conclusions:**

Among patients with stable CAD undergoing DES implantation, long-term maintenance with beta-blocker treatment might not be associated with clinical outcome improvement.

**Trial Registration:**

ClinicalTrial.gov (NCT04715594).

## Introduction

Beta-blockers are considered the primary choice in long-term maintenance drug therapy in patients with coronary artery disease, based on positive evidence for improving clinical outcomes in patients with acute myocardial infarction (MI) (1, 2) or heart failure (3). Long-term beta-blocker maintenance is associated with reduced mortality after percutaneous coronary intervention (PCI) in patients with acute MI (4). However, there is a lack of evidence supporting the beneficial impact of long-term beta-blocker treatment in patients with stable coronary artery disease (CAD) (5). Randomized clinical studies, which usually enroll small patient numbers (6), and observational studies (7, 8) have found no significant benefit to beta-blocker treatment in reducing mortality or ischemic events among patients with stable CAD. Furthermore, published data evaluating the clinical benefits of beta-blocker treatment in patients with stable CAD under the specific situation of post-PCI with drug-eluting stents (DES) is very rare. Using a nationwide cohort database, we sought to investigate the clinical impact of long-term beta-blocker maintenance in patients with stable CAD after PCI with DES.

## Materials and Methods

### Study Design and Data

This study was a nationwide retrospective analysis of the National Health Claims database established by the National Health Insurance Service (NHIS) of Korea, which contains claimed medical costs, detailed information on prescribed drugs including the number of pills and drug dosage, and medical history presented as International Classification of Diseases, Tenth Revision (ICD-10) codes. A majority (97.1%) of the Korean population is required to subscribe to the NHIS, which is the sole insurer managed by the Korean government. Considering that the NHIS also covers information for the remaining population (2.9%) categorized as medical aid subjects, this cohort is considered to represent the entire Korean population (9). This study was approved by the Institutional Review Board of our institute. Informed consent was waived because personal information was masked after cohort generation according to strict confidentiality guidelines of the Korean Health Insurance Review and Assessment Service. This study is registered at ClinicalTrial.gov (NCT04715594). We were also provided with death certificates including ICD-10 codes from the National Institute of Statistics of Korea.

### Study Population

**Figure 1:**
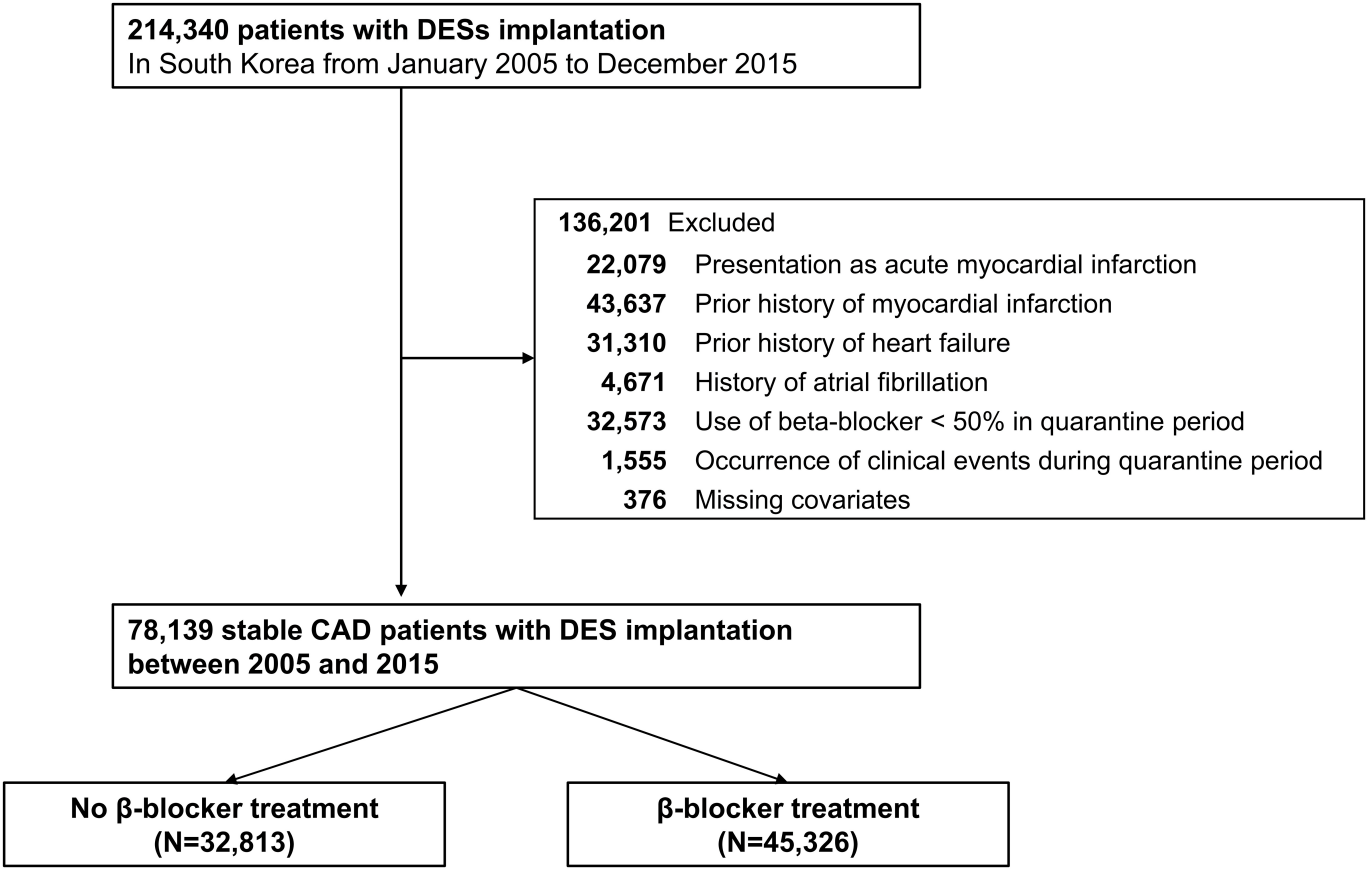
Flow chart of the study population. CAD, coronary artery disease; DES, drug-eluting stent.

Among the 52 million citizens included in the NHIS database, we identified 214,340 patients (≥20 years old) who underwent DES implantation between January 2005 and December 2015, in Korea (CONNECT DES cohort registry). Patients with current (*n* = 22,079) or prior (*n* = 43,637) history of MI, history of heart failure (*n* = 31,310), or history of atrial fibrillation (*n* = 4,671) were excluded from this study. Furthermore, patients with missing covariates were excluded (*n* = 376). Patients with an insufficient period of beta-blocker prescription (<90 days, *n* = 32,573) or those with any clinical event (*n* = 1,555) during 180 days of quarantine were also excluded from the analyses. Consequently, the remaining 78,139 patients with stable CAD that was treated with DES implantation were included in the analysis of this study (Figure 1).

### Study Procedures and Outcomes

To emulate a randomized clinical trial that compares the impact of long-term beta-blocker treatment in patients with stable CAD, we used an intention-to-treat design for beta-blocker treatment, defined as a prescription of more than a 90-day supply of beta-blocker during 180 days of quarantine since index PCI. Types of prescribed beta-blockers are presented in Supplementary Table 1. We utilized ICD-10 codes, fee-for-service, and prescribed medication codes provided by the NHIS database and death certificates provided by the National Statistical Office. The primary outcome of interest was major adverse cardiovascular events (MACE) composed of cardiovascular death, MI, and hospitalization with heart failure for 5 years after PCI with 6 months of quarantine. Secondary outcomes were all-cause death and the individual MACE components. Cardiovascular death was ascertained from the National Statistical Office of Korea, which provided death certificates with an accuracy of 92% for the specific causes of death (9–11). Cardiovascular death was identified by a death certificate with at least one cardiovascular-related diagnosis (acute MI, stroke, heart failure, or sudden cardiac death). MI was defined by the ICD-10 codes corresponding to acute MI (10) and satisfaction of one or more of the following conditions: (1) concurrent presence of claims for coronary angiography, (2) admission *via* the emergency department, or (3) cardiac biomarkers tested more than 4 times. A detailed description of each clinical outcome, including the definition of hospitalization due to heart failure, is presented in Supplementary Table 2. Additionally, we included baseline comorbidities and drug prescription status before PCI for propensity score calculation, and stabilized inverse probability of treatment weights (IPTW) was used to accounting for differences in baseline characteristics, medical history, and confounding bias (Supplementary Table 3).

### Statistical Analysis

Continuous variables are reported as mean and SD, and dichotomous variables are presented as frequency and percentage. To minimize the effect of confounding bias, we calculated the IPTW using the propensity score, which was calculated by logistic regression with covariates of age, sex, history of comorbidities and medications, and year of PCI (Supplementary Table 4). We also stabilized IPTW by multiplying it by the marginal probability of receiving treatment. The effect size difference between the two groups for all comorbidities and medications was calculated using standardized mean difference and Kernel density plots. Standardized mean difference values above 0.2 were regarded as a potential imbalance between the two groups. Cumulative incidence curves and the rate of clinical outcomes of interest during follow-up were plotted using the Kaplan–Meier method. The adjusted hazard ratio (HR) for each clinical outcome of interest was calculated using a Cox proportional hazard regression model. A cause-specific hazard model was used to consider death as a competing risk when comparing the incidences of cardiovascular death, MI, and hospitalization due to heart failure. We further conducted sensitivity analyses to assess the robustness of the main results. First, the heterogeneity of treatment effects in subgroups was assessed using interaction terms in a Cox proportional hazard model. Second, to estimate the effect of continuous treatment, the rank-preserving structural failure times (RPSFT) model was used (12). This method estimates counterfactual event times that would have occurred if patients had not switched treatments (13). It also uses a counterfactual framework to estimate the common causal effect of the treatment using a grid search method and may be associated with low bias when a large number of patients switch treatments (14, 15). Since the RPSFT model was designed originally for analysis of a randomized controlled trial with frequent the crossover between treatment groups (15), our observational study utilized the RPSFT model after propensity score-matching to establish homogenous covariate balance at baseline between patients treated with and without beta-blocker (Supplementary Table 4). Third, we performed a time-varying Cox regression in which treatment (with or without beta-blocker) was a time-dependent variable considering switch between treatments in real-world practice. Among the patients who were assigned to the beta-blocker treatment group during a quarantine period, those with discontinuation of beta-blocker for ≥90 days were considered unexposed during the interval. Fourth, we conducted an intention-to-treat analysis by assigning patients treated with beta-blockers for more than 1 day during the quarantine period, instead of 90 days, to the treatment group. Fifth, we defined the intention-to-treat group as a prescription of more than a 16-day supply of beta-blocker in the 30-day quarantine period after PCI because the 180-day observational period used in the main analysis could have masked the occurrence of adverse clinical events early after DES implantation.

All tests were two-sided and a *p*-value < 0.05 was considered statistically significant. Statistical analyses were conducted using SAS version 9.4 (SAS Institute, Cary, NC, USA) and R version 3.6 with “RPSFTM” and “survival” packages (The R Foundation, www.R-project.org).

## Results

**Figure 2:**
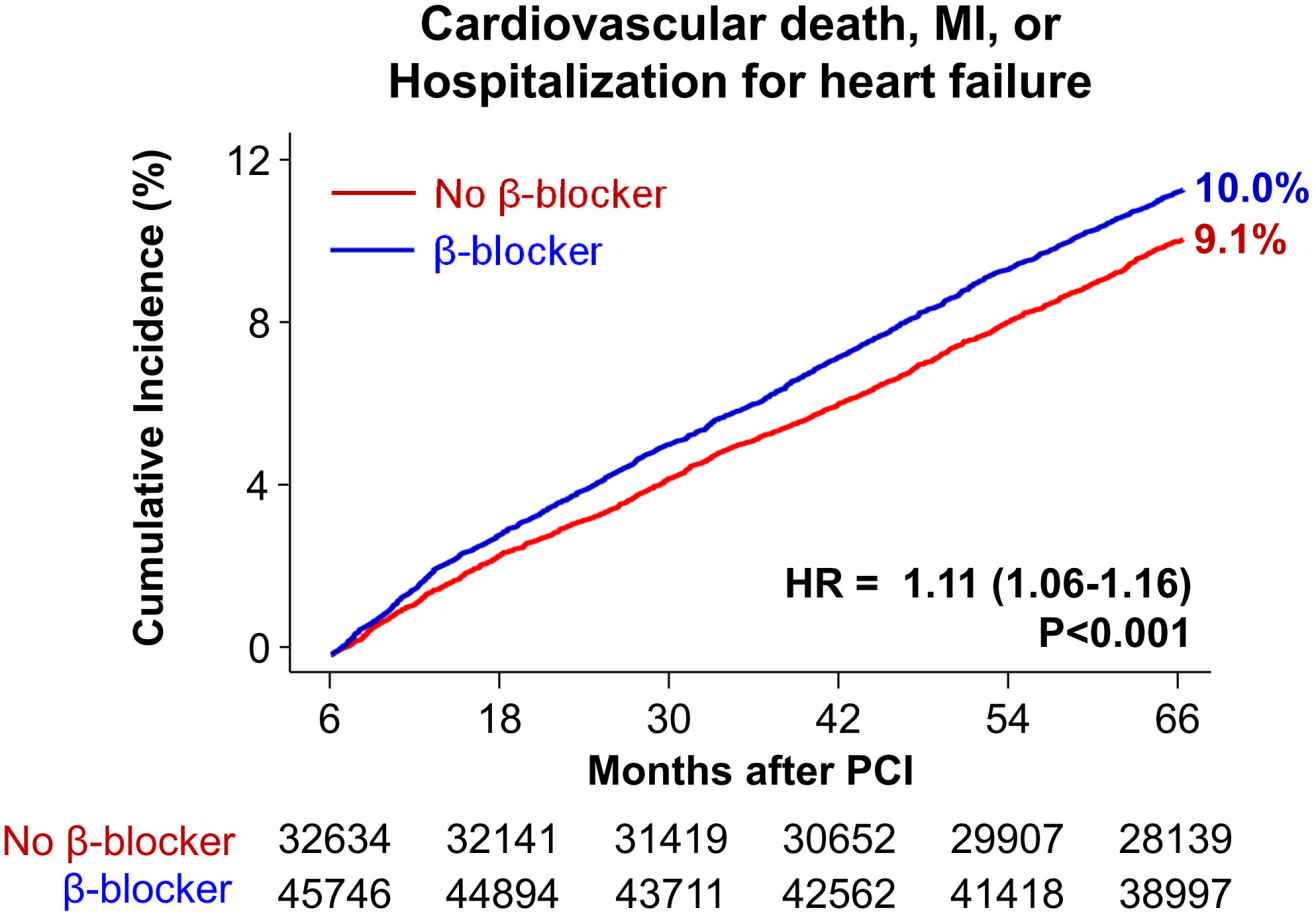
Time-to-event curves for major adverse cardiovascular events for 5 years after PCI. The cumulative incidence of major adverse cardiovascular events for 5 years after PCI. HR, hazard ratio; MI, myocardial infarction; PCI, percutaneous coronary intervention.

**Figure 3:**
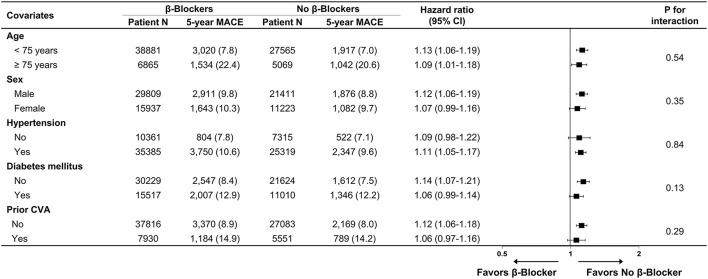
Subgroup analysis for major adverse cardiovascular events. Numbers and percentages show the number of patients at risk, number of events, and the incidence rate of major adverse cardiac events 5 years after drug-eluting stent implantation. CI, confidence interval; CVA, cerebrovascular accidents; MACE, major adverse cardiovascular events.

**Figure 4:**
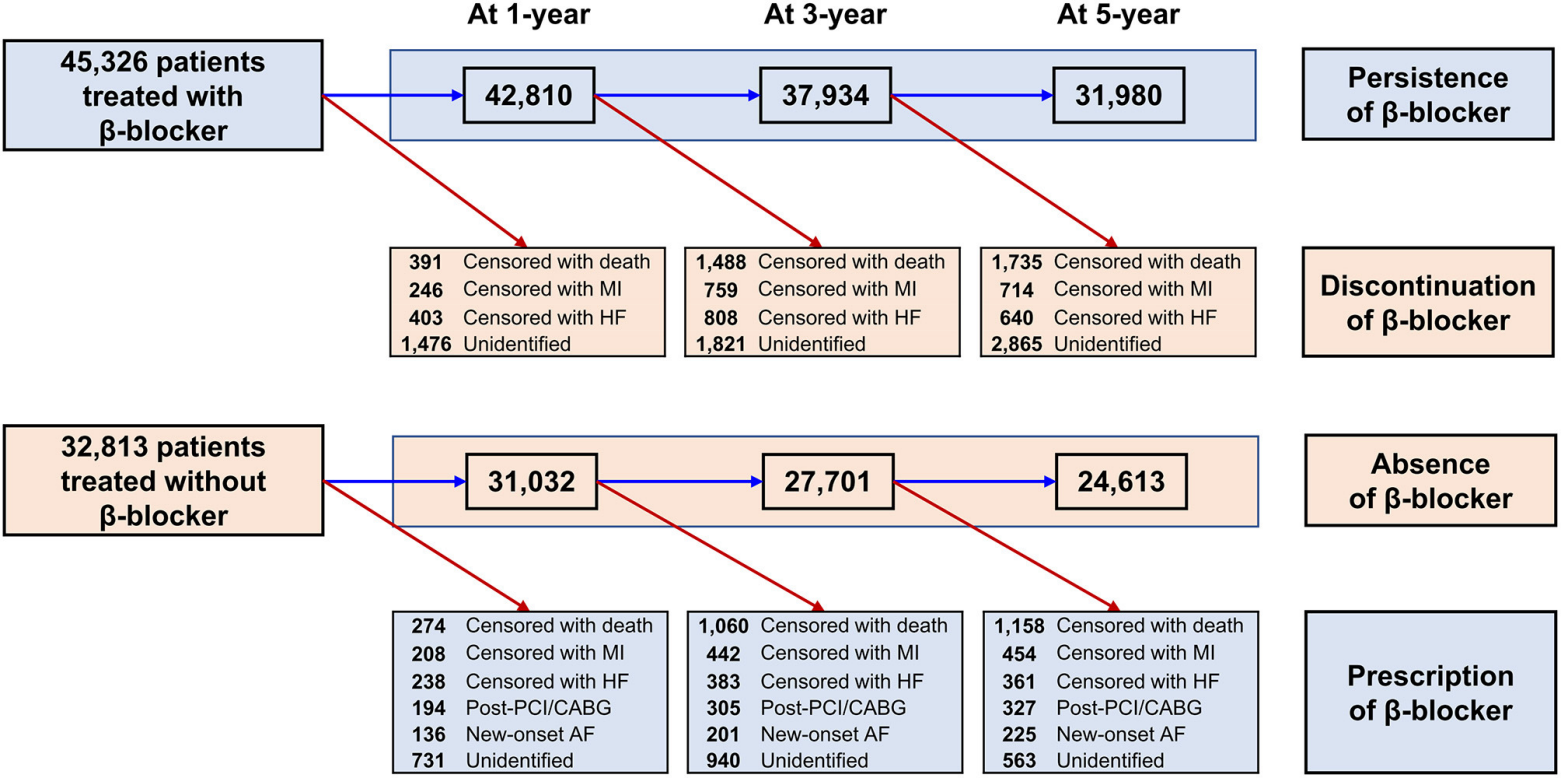
Temporal trends in change of beta-blocker prescription. AF, atrial fibrillation; CABG, coronary artery bypass graft surgery; HF, heart failure; MI, myocardial infarction; PCI, percutaneous coronary intervention.

**Figure 5:**
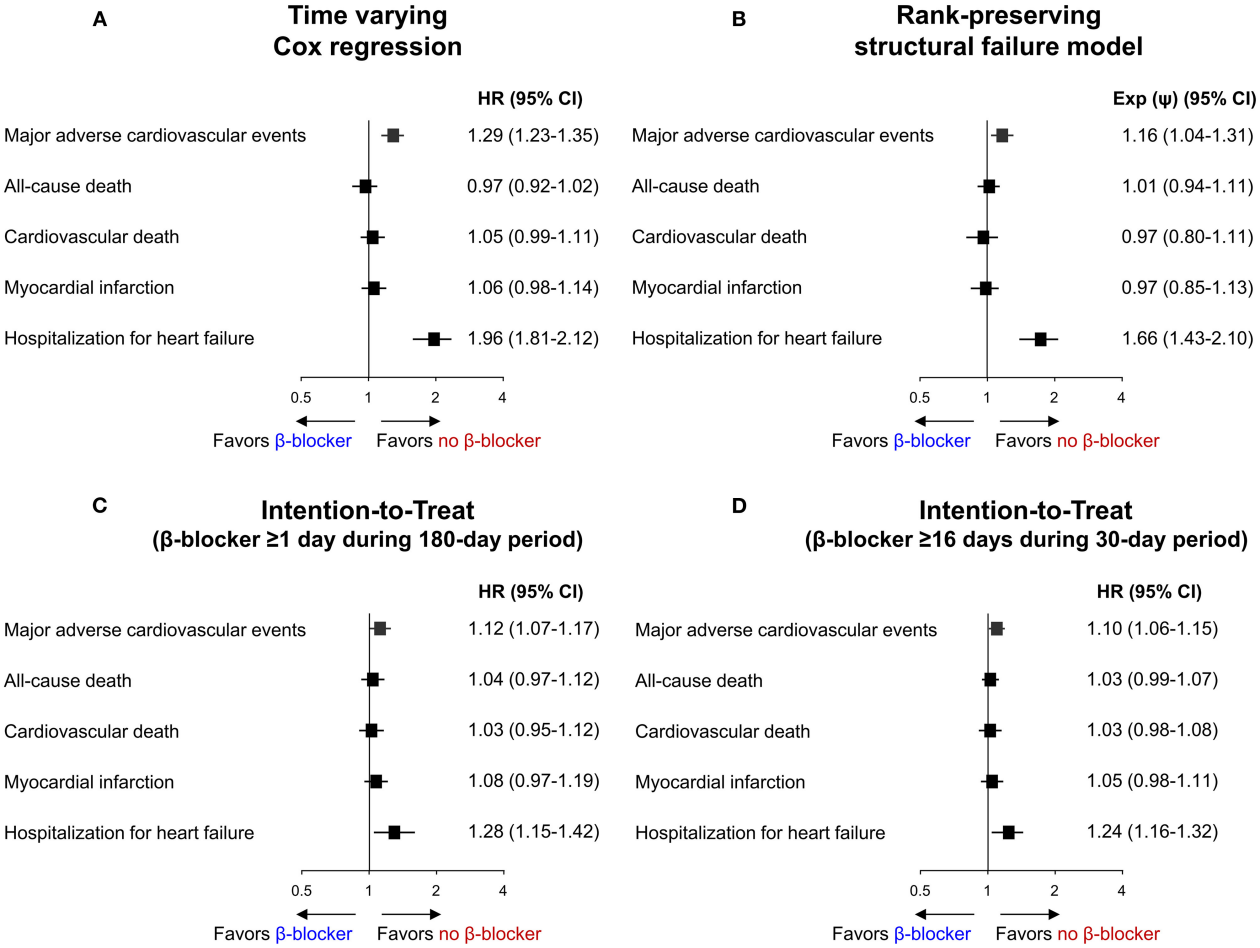
Sensitivity analysis for primary and secondary outcomes. Risk of primary and secondary outcomes according to beta-blocker treatment analyzed by **(A)** Time varying Cox regression, **(B)** Rank-preserving structural failure model, and **(C,D)** Intention-to-Treat method. CI, confidence interval; HR, hazard ratio. Exp (ψ) indicates an increase/decrease in survival in the non-treatment group.

Baseline demographics and medical history of the cohort population before and after stabilized IPTW are presented in Table 1. After weighting, 78,380 DES-treated patients were included: 45,746 with and 32,634 without beta-blocker treatment. After stabilized IPTW, there was no evidence of inequality in baseline demographic characteristics or medical history between the two groups (all standardized mean difference <0.1, Supplementary Figures 1, 2). The incidence and relative hazards of primary and secondary outcomes are presented in Table 2, Figure 2, and Supplementary Figure 3. At 5 years after PCI with 6 months of quarantine, the adjusted incidence rate of MACE was significantly higher in patients treated with beta-blockers (10.0 vs. 9.1% in those without beta-blocker treatment; HR 1.11, 95% CI 1.06–1.16, *p* < 0.001, Figure 2) in an intention-to-treat analysis (Table 2 and Supplementary Table 5). There was no significant difference in all-cause death between patients treated with and without beta-blocker (8.1 vs. 8.2%; HR 0.99, 95% CI 0.94–1.04, *p* = 0.62, Supplementary Figure 3), As for the individual components of MACE, there was no significant association between beta-blocker treatment and risk of cardiovascular death (5.9 vs. 5.9% in those without beta-blocker treatment; HR 1.00, 95% CI 0.94–1.06, *p* = 0.88) or MI (3.8 vs. 3.6% in those without beta-blocker treatment; HR 1.03, 95% CI 0.96–1.11, *p* = 0.42), while the adjusted hospitalization rate due to heart failure was significantly higher in patients treated with beta-blockers (4.1 vs. 3.1% in those without beta-blocker treatment; HR 1.32, 95% CI 1.23–1.43, *p* < 0.001) (Table 2). Consistent findings were observed regardless of DAPT duration (Supplementary Table 6). In a subgroup analysis, there was no significant interaction between the baseline comorbidities and beta-blocker treatment for a 5-year occurrence of MACE (Figure 3) or all-cause mortality (Supplementary Figure 4). There was no significant difference in treatment effect according to the generation of beta-blockers (Supplementary Table 7). During a 5-year follow-up period, there were frequent changes in the prescribed beta-blocker status during follow-up (Figure 4). Among 45,326 patients initially treated with beta-blocker before stabilized IPTW weighting, administration of beta-blocker was discontinued in 6,162 (13.6%) without adverse cardiovascular events such as death, MI, or heart failure. Of the 32,813 patients initially treated without beta-blocker, 3,622 (11.0%) started taking beta-blocker during the 5-year follow-up period and showed no MI or heart failure. To take into account frequent cross-over between the treatment groups, we performed additional statistical analyses with time-varying Cox regression (Figure 5A) and RPSFT models (Figure 5B), which demonstrated no statistically significant impact of beta-blocker treatment on the occurrence of all-cause death, cardiovascular death, or MI, whereas beta-blocker treatment was associated with a higher incidence rate of MACE or hospitalization for heart failure. Consistent findings were obtained when patients treated with beta-blocker for more than 1 day during 180 days of quarantine were considered as a treatment group (Figure 5C) or when the prescription status of beta-blocker within a 30-day period, instead of a 180-day quarantine period, after index PCI was applied (Figure 5D).

**Table 1:** Baseline characteristics and medications in all patients.

**Characteristics**	**Before stabilized IPTW (*****N*** **=** **78,139)**			**After stabilized IPTW (*****N*** **=7 8,380)**		
	**No β-blocker (*N* = 32,813)**	**β-blocker (*N* = 45,326)**	**SMD**	**No β-blocker (*N* = 32,634)**	**β-blocker (*N* = 45,746)**	**SMD**
Age, years	63.6 ± 10.2	63.8 ± 10.1	0.021	63.8 ± 10.2	63.8 ± 10.2	0.008
Female	10,109 (30.8)	16,460 (36.3)	0.117	11,223 (34.4)	15,937 (34.8)	0.009
**Comorbidity**						
Hypertension	23,651 (72.1)	36,785 (81.2)	0.216	25,319 (77.6)	35,385 (77.4)	0.006
Dyslipidemia	18,751 (57.1)	23,619 (52.1)	0.101	17,721 (54.3)	24,791 (54.2)	0.002
Chronic kidney disease with severe renal impairment^*^	1,177 (3.6)	2,510 (5.5)	0.094	1,636 (5.0)	2,159 (4.7)	0.014
Diabetes mellitus	10,490 (32.0)	15,752 (34.8)	0.059	11,010 (33.7)	15,517 (33.9)	0.004
Chronic liver disease	8,587 (26.2)	10,584 (23.4)	0.065	8,029 (24.6)	11,229 (24.5)	0.001
Chronic pulmonary disease	9,969 (30.4)	12,215 (26.9)	0.076	9,445 (28.9)	13,202 (28.9)	0.002
Peripheral arterial occlusive disease	2,442 (7.4)	2,911 (6.4)	0.040	2,289 (7.0)	3,259 (7.1)	0.004
Prior malignancy	2,384 (7.3)	3,009 (6.6)	0.025	2,277 (7.0)	3,159 (6.9)	0.003
Prior stroke or TIA	5,350 (16.3)	7,447 (16.4)	0.003	5,551 (17.0)	7,930 (17.3)	0.009
Prior ICH	300 (0.9)	448 (1.0)	0.008	321 (1.0)	473 (1.0)	0.005
Prior PCI or CABG	639 (1.8)	957 (2.1)	0.007	624 (1.9)	933 (2.0)	0.005
Osteoporosis	4,917 (15.0)	6,769 (14.9)	0.001	4,977 (15.3)	7,042 (15.4)	0.004
Thyroid disorder	1,837 (5.6)	2,249 (5.0)	0.028	1,691 (5.2)	2,393 (5.2)	0.002
Charlson comorbidity index	2.6 ± 2.1	2.5 ± 2.2	0.047	2.6 ± 2.2	2.5 ± 2.2	0.011
**Medication before PCI**						
Aspirin	18,133 (55.3)	27,769 (61.3)	0.122	18,876 (57.8)	26,089 (57.0)	0.016
Clopidogrel	11,657 (35.5)	16,285 (35.9)	0.008	11,348 (34.8)	15,574 (34.0)	0.015
β-Blocker	9,440 (28.8)	42,527 (93.8)	1.794	21,635 (66.3)	30,126 (65.9)	0.009
RAAS blockade	16,090 (49.0)	29,474 (65.0)	0.327	18,817 (57.7)	26,400 (57.7)	0.001
**Procedural information**						
Number of stents	1.2 ± 0.4	1.2 ± 0.4	0.022	1.2 ± 0.4	1.2 ± 0.4	0.002
Type of DES						
First-generation DES^†^	6,779 (20.7)	15,004 (33.1)	0.283	8,797 (27.0)	12,254 (26.8)	0.004
Next-generation DES	26,034 (79.3)	30,322 (66.9)		23,837 (73.0)	33,492 (73.2)	
DAPT duration post-PCI, days	907.3 ± 581.9	934.8 ± 577.8	0.047	913.6 ± 582.1	933.5 ± 577.7	0.032
**Year of PCI**						
2005	1,752 (5.3)	4,839 (10.7)	0.416	2,562 (7.8)	3,702 (8.1)	0.019
2006	1,918 (5.8)	4,327 (9.5)		2,484 (7.6)	3,458 (7.6)	
2007	1,274 (3.9)	2,916 (6.4)		1,721 (5.3)	2,367 (5.2)	
2008	1,876 (5.7)	3,594 (7.9)		2,221 (6.8)	3,026 (6.6)	
2009	2,305 (7.0)	4,421 (9.8)		2,787 (8.5)	3,789 (8.3)	
2010	2,831 (8.6)	5,003 (11.0)		3,301 (10.1)	4,522 (9.9)	
2011	2,947 (9.0)	3,678 (8.1)		2,865 (8.8)	4,027 (8.8)	
2012	2,737 (8.3)	2,942 (6.5)		2,438 (7.5)	3,445 (7.5)	
2013	3,091 (9.4)	3,256 (7.2)		2,734 (8.4)	3,886 (8.5)	
2014	5,479 (16.7)	4,993 (11.0)		4,443 (13.6)	6,335 (13.8)	
2015	6,603 (20.1)	5,357 (11.8)		5,078 (15.6)	7,192 (15.7)	

*Values are presented as the mean ± SD or n (%).IPTW, inverse probability of treatment weighting; SMD, standardized mean difference; TIA, transient ischemic attack; ICH, intracranial hemorrhage; PCI, percutaneous coronary intervention; CABG, coronary artery bypass graft; DES, drug-eluting stent; DAPT, dual antiplatelet therapy; RAAS, renin-angiotensin-aldosterone-system*.

* *Chronic kidney disease with advanced stage requiring intensive medical therapy and financial assistance from health insurance*.

† *First-generation drug-eluting stent indicates Cypher and Taxus*.

**Table 2:** Risks of primary and secondary outcomes at 5 years after percutaneous coronary intervention between patients prescribed with or without β-blocker after stabilized inverse probability of treatment weighting.

	**No β-blocker (*N* = 32,634)**	**β-blocker (*N* = 45,746)**	**Risk difference (95% CI)**	**Hazard ratio (95% CI)**	***p-*value**
Major adverse cardiovascular event^*^	2,958 (9.1)	4,554 (10.0)	0.9 (0.5 to 1.3)	1.11 (1.06–1.16)	<0.001
All-cause death	2,688 (8.2)	3,722 (8.1)	−0.1 (-0.5 to 0.3)	0.99 (0.94–1.04)	0.62
Cardiovascular death	1,934 (5.9)	2,697 (5.9)	0.0 (-0.4 to 0.3)	1.00 (0.94–1.06)	0.88
Myocardial infarction	1,189 (3.6)	1,717 (3.8)	0.1 (-0.2 to 0.4)	1.03 (0.96–1.11)	0.42
Hospitalization for heart failure	1,018 (3.1)	1,879 (4.1)	1.0 (0.7 to 1.3)	1.32 (1.23–1.43)	<0.001

* *Composite of cardiovascular death, myocardial infarction, and hospitalization for heart failure*.

## Discussion

This nationwide cohort analysis evaluated the association between long-term beta-blocker treatment and clinical outcomes including mortality in patients with stable CAD after DES implantation. Taking advantage of the unique feature of the Korean NHIS database that accurately tracks all medication information over the entire study period, we could analyze the clinical impact of long-term beta-blocker administration in real-world practice by emulating the intention-to-treat manner of a randomized controlled trial. Furthermore, we applied time-varying Cox regression analysis and the RPSFT model to account for switching between treatment strategies, which is typical in real-world practice. The major findings of our study are as follows: (1) in patients with stable CAD after DES implantation, long-term maintenance treatment with beta-blockers was not associated with improvement of clinical outcomes, and (2) sensitivity analyses that considered switching between treatment strategies revealed consistent findings of a negligible impact of beta-blocker on clinical outcomes.

Long-term maintenance beta-blocker treatment after PCI in patients with MI or heart failure is recommended highly based on a large body of evidence that the treatment reduces mortality and morbidity (4, 16). This benefit mainly relies on the heart rate lowering property that decreases oxygen requirements, and negative inotropic effects that mitigate adverse cardiac remodeling and ventricular arrhythmia (17). Furthermore, in a COURAGE (Clinical Outcomes Utilizing Revascularization and Aggressive Drug Evaluation) trial, which demonstrated comparable effects of optimal medical therapy to PCI in stable CAD patients, beta-blockers were a mainstay drug treatment prescribed in 87% of patients enrolled in the trial (18).

There are two published studies that evaluated the association between beta-blocker treatment at discharge and clinical outcomes in stable CAD patients undergoing PCI without prior history of MI or heart failure. One registry study (*n* = 5,288) reported that beta-blocker treatment at discharge was associated with a significantly increased risk of cardiac death/MI during a 3-year follow-up after index PCI (HR 1.48, 95% CI 1.05–2.10, *p* =0.02) (19). Another registry study with a larger number of patients (*n* = 122,734) reported no significant association between beta-blocker treatment at discharge and mortality or MI at 3-year follow-up (8). In addition, discharge with beta-blocker treatment was associated with more frequent readmission due to heart failure (8). However, these two studies did not provide detailed information on the prescribed beta-blocker status during the 3-year follow-up (8, 19). Because continuous beta-blocker prescription status after discharge during long-term follow-up was not evaluated clearly, the impact of drug switch during the follow-up period was not addressed (8, 19).

To minimize potential sources for bias, we excluded patients with concomitant indications for beta-blocker treatment such as MI, heart failure, or atrial fibrillation. Next, we emulated randomized controlled trials using intention-to-treat analysis with a 180-day quarantine period to assign treatment groups and the stabilized IPTW model to adjust for baseline differences. Furthermore, taking advantage of the unique strength of the NHIS database of Korea, which enables tracking and tracing of complete medication information during an entire follow-up period, we compared the main results with those of sensitivity analyses using the time-varying Cox regression and RPSFT model, which consider switches between treatment groups during follow-up. Finally, to compensate for the possible immortal time bias caused by 6 months of quarantine without clinical events, we set a quarantine period of 1 month as a sensitivity analysis. Results from observational studies cannot be used to establish causality, and residual perturbations can persist after propensity score weighting. However, despite the heterogeneity of treatment groups, various sensitivity analyses confirmed consistency compared with the main analysis.

In our analysis, 14% of patients initially treated with beta-blocker after DES implantation eventually discontinued the drug without the occurrence of significant clinical events. In fact, patients who are prescribed beta-blocker can complain of numerous side effects such as fatigue, bradycardia, depression, hypotension, bronchospasm, peripheral vasoconstriction, or postural hypotension, which usually leads to discontinuation of beta-blocker treatment. Furthermore, chronic beta-blocker use has been associated with lipid profile deterioration and new-onset diabetes (20–22). One study reported that beta-blocker treatment increased serum triglyceride level, decreased HDL cholesterol level, and increased plasma small dense LDL, resulting in an atherogenic lipoprotein phenotype (23). A meta-analysis that included 94,492 hypertensive patients treated with beta-blocker has suggested a positive association between beta-blocker treatment and new-onset diabetes (21). Furthermore, non-selective beta-blockers can cause coronary artery spasms by inhibiting β-adrenergic mediated vasodilation (24). Concerns for possible side effects of long-term beta-blocker administration weaken the rationale for routine use of beta-blocker in specific patients with stable CAD after PCI with DES.

## Limitations

This study has several limitations. First, in this nationwide cohort based on claims data, the systolic function of the left ventricle before PCI and during the follow-up period are not included; thus, patients with borderline left ventricular systolic function or reduced left ventricular systolic function without heart failure diagnosis could be included. Furthermore, in the administrative database, heart failure with preserved ejection fraction could have been underdiagnosed (25). Second, considering the nature of retrospective data based on claims, the findings presented in this study cannot be used to establish causal associations, and residual confounding variables could persist even after stabilized IPTW. Third, we adopted the RPSFT model to correct for frequent changes in beta-blocker prescription. Since the RPSFT is a statistical model typically applied to the analysis of randomized controlled trials, caution is needed in interpreting the findings obtained through this analysis despite the 1:1 propensity score matching for the establishment of RPSFT.

## Conclusions

Among patients with stable CAD undergoing DES implantation, long-term maintenance with beta-blocker treatment was associated with an increased occurrence of MACE. Beta-blocker treatment may not be recommended as a maintenance drug therapy in specific patients with stable CAD after index PCI.

## Data Availability Statement

The datasets generated for the analyses are not publicly available because of strict government restrictions. Requests to access these datasets should be directed to M-KH, mkhong61@yuhs.ac.

## Ethics Statement

The studies involving human participants were reviewed and approved by Yonsei University Health System Institutional Review Board. The Ethics Committee waived the requirement of written informed consent for participation.

## Author Contributions

S-JL, D-WC, YS, CK, S-JH, C-MA, J-SK, B-KK, Y-GK, DC, E-CP, YJ, C-MN, and M-KH contributed to the conception and design. S-JL and M-KH wrote the study protocol. D-WC and C-MN performed the programming to extract the data from the NHIS database. C-MN and M-KH had full access to all the data in the study and take responsibility for the integrity of the data and the accuracy of the data analysis. S-JL, D-WC, C-MN, and M-KH verified the data and conducted all analyses. YS, CK, S-JH, C-MA, J-SK, B-KK, Y-GK, DC, E-CP, and YJ provided a critical review of the manuscript. All authors read and approved the final publication.

## Funding

This work was supported by the Cardiovascular Research Center, Seoul, Korea.

## Conflict of Interest

The authors declare that the research was conducted in the absence of any commercial or financial relationships that could be construed as a potential conflict of interest.

## Publisher's Note

All claims expressed in this article are solely those of the authors and do not necessarily represent those of their affiliated organizations, or those of the publisher, the editors and the reviewers. Any product that may be evaluated in this article, or claim that may be made by its manufacturer, is not guaranteed or endorsed by the publisher.

## Abbreviations

CAD: coronary artery disease
CI: confidence interval
DES: drug-eluting stent
HR: hazard ratio
IPTW: inverse probability of treatment weights
MACE: major adverse cardiovascular events
MI: myocardial infarction
NHIS: national health insurance service
PCI: percutaneous coronary intervention
RPSFT: rank-preserving structural failure times.
