# Supplementary material for: Long-Term Beta-Blocker Therapy in Patients With Stable Coronary Artery Disease After Percutaneous Coronary Intervention
Source: Front Cardiovasc Med. 2022 May 17;9:878003. doi: 10.3389/fcvm.2022.878003 (PMC9152083; doi:10.3389/fcvm.2022.878003)
Supplement: Supplementary file 1 [file Data_Sheet_1.docx]

**SUPPLEMENTAL MATERIAL ONLINE**

**Long-Term Beta-Blocker Therapy in Patients with Stable Coronary Artery Disease after Percutaneous Coronary Intervention**

Seung-Jun Lee, Dong-Woo Choi, Yongsung Suh, Sung-Jin Hong, Chul-Min Ahn, Jung-Sun Kim, Byeong-Keuk Kim, Young-Guk Ko, Donghoon Choi, Eun-Cheol Park, Yangsoo Jang, Chung-Mo Nam, Myeong-Ki Hong

| **Contents** | **Page** |
| --- | --- |
| **Table 1.** Type of β-blocker prescribed during quarantine period | 2 |
| **Table 2.** Individual outcome definitions | 3 |
| **Table 3.** Covariates include in stabilized inverse probability of treatment weighting | 4 |
| **Table 4.** Baseline characteristics and medications before and after propensity score matching for rank-preserved structure failure time model | 5 |
| **Table 5.** Risks of primary and secondary outcomes at 5 years after PCI between patients prescribed with or without β-blocker after stabilized inverse probability of treatment weighting | 7 |
| **Table 6.** Risks of primary and secondary outcomes at 5 years after PCI between patients prescribed with or without β-blocker according to the DAPT duration | 8 |
| **Table 7.** Risks of primary and secondary outcomes at 5 years after PCI between patients prescribed with conventional or third-generation β-blockers. | 9 |
| **Figure 1.** Standardized difference before and after stabilized inverse probability of treatment weighting | 10 |
| **Figure 2.** Distribution of stabilized inverse probability of treatment weightings | 11 |
| **Figure 3.** Time-to-event curves for all-cause death for 5 years after PCI | 12 |
| **Figure 4.** Subgroup analysis for all-cause death | 13 |

**Supplemental Table 1.** Type of β-blockers prescribed at quarantine period.

|  | **N (%)** |
| --- | --- |
| Carvedilol | 19,431 (40.8) |
| Bisoprolol | 11,954 (25.1) |
| Atenolol | 7,572 (15.9) |
| Nebivolol | 3,810 (8.0) |
| Propranolol | 1,667 (3.5) |
| Metoprolol | 1,048 (2.2) |
| Bevantolol | 667 (1.4) |
| Labetalol | 620 (1.3) |
| Betaxolol | 618 (1.3) |
| Arotinolol | 190 (0.4) |
| Cartenolol | 48 (0.1) |

**Supplemental Table 2**. **Individual outcome definitions**

| **Outcomes** | **Logical description** |
| --- | --- |
| Cardiovascular mortality | - Cardiac death confirmed by death certificate - Death with ICD-10 codes corresponding to coronary artery disease, acute MI, heart failure or stroke within 1 month |
| Myocardial infarction (MI) | - ICD-10 codes corresponding to acute MI (I21, I22, I25.2) - Performance of coronary angiography within 7 days - Admission via emergency department - Performance of cardiac enzyme check for more than 4 times |
| Hospitalization for heart failure | - ICD-10 codes corresponding to heart failure (I11.0, I50, I97.1) - Admission for more than 3 days |

**Supplemental Table 3. Covariates include in stabilized inverse probability of treatment weighting**

|  | **Variable list** |
| --- | --- |
| **Baseline characteristics** | Age, gender, diabetes, hypertension, dyslipidemia, history of heart failure, presentation as acute myocardial infarction, prior history of stroke or cerebrovascular accident, prior history of intracranial hemorrhage, atrial fibrillation or flutter, prior history of malignancy, hyperthyroidism, hypothyroidism, osteoporosis, chronic liver disease, chronic pulmonary disease, chronic kidney disease with severe renal impairment, type of drug-eluting stent (1^st^-generation versus next-generation). |
| **Medications*** | Warfarin sodium, Edoxaban, Rivaroxaban, Apixaban, Dabigatran, Aspirin, Clopidogrel, Prasugrel, Ticagrelor, Atorvastatin, Rosuvastatin, Simvastatin, Pravastatin, Fluvastatin, Pitavastatin, Lovastatin, Tripamol, Hydrochlorothiazide, Chlorthalidon, Indapamide, Metorazone, Furosemide, Torasemide, Amlodipine, Barnidipine, Cilnidipine, Felodipine, Lacidipine, Lercanidipine, Manidipine, Nicardipine, Nifedipine, Nisoldipine, Nitrendipine, Diltiazem, Verapamil, Bunazosin, Doxazosin, Prazosin, Terazosin, Sulfonylurea, Metformin, Alpha-glucosidase inhibitors, Thiazolidinedione, DPP-Ⅳ inhibitors, SGLT-2 inhibitors, insulin, Spironolactone, Alacepril, Benazepril, Captopril, Cilazapril, Delapril, Enalapril, Fosinopril, Imidapril, Lisinopril, Moexipril, Perindopril, Quinapril, Ramipril, Temocapril, Candesartan, Eprosartan, Irbesartan, Losartan, Telmisartan, Valsartan, Fimasartan, Azilsartan |

*** Prescription during 1-year prior to index percutaneous coronary intervention**

**Supplemental Table 4. Baseline characteristics and medications before and after propensity score matching for rank-preserved structure failure model**

| **Characteristics** | | **Before PS matching (N=78,139)** | | | **After 1:1 PS matching (N=24,480)** | | |
| --- | --- | --- | --- | --- | --- | --- | --- |
|  | **No β-blocker**  **(N=32,813)** | | **β-blocker**  **(N=45,326)** | **SMD** | **No β-blockers**  **(N=12,240)** | **β-blockers**  **(N=12,240)** | **SMD** |
| Age, years | 63.6±10.2 | | 63.8±10.1 | 0.021 | 63.84±10.19 | 63.80±10.20 | 0.004 |
| Female | 10,109 (30.8) | | 16,460 (36.3) | 0.117 | 4,158 (34.0) | 4,223 (34.5) | 0.011 |
| **Comorbidity** | | | | | | | |
| Hypertension | 23,651 (72.1) | | 36785 (81.2) | 0.216 | 9,423 (77.0) | 9,612 (78.5) | 0.037 |
| Dyslipidemia | 18,751 (57.1) | | 23619 (52.1) | 0.101 | 6,963 (56.9) | 6,812 (55.7) | 0.025 |
| Chronic kidney disease with  severe renal impairment ^a^ | 1,177 (3.6) | | 2510 (5.5) | 0.094 | 526 (4.3) | 573 (4.7) | 0.019 |
| Diabetes Mellitus | 10,490 (32.0) | | 15752 (34.8) | 0.059 | 4,032 (32.9) | 4,113 (33.6) | 0.014 |
| Chronic liver disease | 8,587 (26.2) | | 10584 (23.4) | 0.065 | 3,207 (26.2) | 3,081 (25.2) | 0.024 |
| Chronic pulmonary disease | 9,969 (30.4) | | 12215 (26.9) | 0.076 | 3,760 (30.7) | 3,630 (29.7) | 0.023 |
| Peripheral arterial occlusive disease | 2,442 (7.4) | | 2911 (6.4) | 0.040 | 938 (7.7) | 862 (7.0) | 0.024 |
| Prior malignancy | 2,384 (7.3) | | 3009 (6.6) | 0.025 | 867 (7.1) | 855 (7.0) | 0.004 |
| Prior stroke or TIA | 5,350 (16.3) | | 7447 (16.4) | 0.003 | 2,179 (17.8) | 2,198 (18.0) | 0.004 |
| Prior ICH | 300 (0.9) | | 448 (1.0) | 0.008 | 129 (1.1) | 109 (0.9) | 0.017 |
| Prior PCI or CABG | 639 (1.8) | | 957 (2.1) | 0.007 | 257 (2.0) | 233 (2.0) | 0.002 |
| Osteoporosis | 4,917 (15.0) | | 6769 (14.9) | 0.001 | 2,011 (16.4) | 1,942 (15.9) | 0.015 |
| Thyroid disorder | 1,837 (5.6) | | 2249 (5.0) | 0.028 | 725 (5.9) | 688 (5.6) | 0.013 |
| Charlson comorbidity index |  | |  |  |  |  |  |
| **Medication before PCI** | | | | | | | |
| Aspirin | 18,133 (55.3) | | 27769 (61.3) | 0.122 | 7,385 (60.3) | 7,207 (58.9) | 0.03 |
| Clopidogrel | 11,657 (35.5) | | 16285 (35.9) | 0.008 | 4,752 (38.8) | 4,537 (37.1) | 0.036 |
| β-Blockers | 9,440 (28.8) | | 4,2527 (93.8) | 1.794 | 9,440 (77.1) | 9,441 (77.1) | <0.001 |
| RAAS blockade | 16,090 (49.0) | | 2,9474 (65.0) | 0.327 | 6,795 (55.5) | 7,101 (58.0) | 0.05 |
| **Procedural information** |  | |  |  |  |  |  |
| Number of stents | 1.2±0.4 | | 1.2±0.4 | 0.022 | 1.2±0.4 | 1.2±0.4 | <0.001 |
| Type of DES |  | |  |  |  |  |  |
| First-generation DES ^b^ | 6,779 (20.7) | | 15,004 (33.1) | 0.283 | 3,268 (26.9) | 3,296 (26.9) | 0.002 |
| Next-generation DES | 26,034 (79.3) | | 30,322 (66.9) |  | 8,972 (73.1) | 8,944 (73.1) |  |
| DAPT duration post-PCI, days | 907.3±581.9 | | 934.8±577.8 | 0.047 | 917.3±567.4 | 926.7±567.3 | 0.018 |
| **Year of PCI** | | | | | | | |
| 2005 | 1,752 (5.3) | | 4,839 (10.7) | 0.416 | 720 (5.9) | 880 (7.2) | 0.098 |
| 2006 | 1,918 (5.8) | | 4,327 (9.5) |  | 759 (6.2) | 871 (7.1) |  |
| 2007 | 1,274 (3.9) | | 2,916 (6.4) |  | 519 (4.2) | 609 (5.0) |  |
| 2008 | 1,876 (5.7) | | 3,594 (7.9) |  | 842 (6.9) | 883 (7.2) |  |
| 2009 | 2,305 (7.0) | | 4,421 (9.8) |  | 979 (8.0) | 1042 (8.5) |  |
| 2010 | 2,831 (8.6) | | 5,003 (11.0) |  | 1,274 (10.4) | 1,275 (10.4) |  |
| 2011 | 2,947 (9.0) | | 3,678 (8.1) |  | 1188 (9.7) | 1,122 (9.2) |  |
| 2012 | 2,737 (8.3) | | 2,942 (6.5) |  | 885 (7.2) | 922 (7.5) |  |
| 2013 | 3,091 (9.4) | | 3,256 (7.2) |  | 1044 (8.5) | 993 (8.1) |  |
| 2014 | 5,479 (16.7) | | 4,993 (11.0) |  | 1,885 (15.4) | 1,744 (14.2) |  |
| 2015 | 6,603 (20.1) | | 5,357 (11.8) |  | 2,145 (17.5) | 1,899 (15.5) |  |

Values are presented as the mean ± standard deviation or n (%). Abbreviations: IPTW, inverse probability of treatment weighting; SMD, standardized mean difference; TIA, transient ischemic attack; ICH, intracranial hemorrhage; PCI, percutaneous coronary intervention; CABG, coronary artery bypass graft; DES, drug-eluting stent; DAPT, dual antiplatelet therapy; RAAS, renin-angiotensin-aldosterone-system.

^a^ Chronic kidney disease with advanced stage requiring intensive medical therapy and financial assistance from health insurance.

^b^ First-generation drug-eluting stent indicates Cypher and Taxus.

**Supplemental Table 5. Risks of primary and secondary outcome at 5-years after percutaneous coronary intervention between patients prescribed with or without β-blockers before stabilized inverse probability of treatment weighting.**

|  | **No β-blocker**  **(N=32,813)** | **β-blocker**  **(N=45,326)** | **Risk difference**  **(95% CI)*** | **Hazard ratio**  **(95% CI)**^†^ | **p-value** |
| --- | --- | --- | --- | --- | --- |
| All-cause death | 2,492 (7.6%) | 3,614 (8.0%) | 0.4 (0.0 to 0.8) | 1.05 (1.00-1.11) | 0.060 |
| Major adverse cardiac event * | 2,738 (8.3%) | 4,437 (9.8%) | 1.4 (1.0 to 1.8) | 1.18 (1.13-1.24) | <0.001 |
| Cardiovascular death | 1,718 (5.2%) | 2,606 (5.7%) | 0.5 (0.2 to 0.8) | 1.10 (1.04-1.17) | 0.003 |
| Myocardial infarction | 1,104 (3.4%) | 1,719 (3.8%) | 0.4 (0.1 to 0.7) | 1.13 (1.05-1.22) | 0.002 |
| Hospitalization for heart failure | 972 (3.0%) | 1,851 (4.1%) | 1.1 (0.8 to 1.4) | 1.39 (1.28-1.50) | <0.001 |

*Composite of cardiovascular death, myocardial infarction, and hospitalization for heart failure.

**Supplemental Table 6. Risks of primary and secondary outcomes at 5 years after percutaneous coronary intervention between patients prescribed with or without β-blocker according to the DAPT duration**

|  | **No β-blocker** | **β-blocker** | **Risk difference**  **(95% CI)** | **Hazard ratio**  **(95% CI)** | **p*-*value** |
| --- | --- | --- | --- | --- | --- |
| **DAPT duration < 12 months** | **(N=6,315)** | **(N=7,747)** |  |  |  |
| Major adverse cardiovascular event* | 854 (13.5) | 1,146 (14.8) | 1.2 (0.5 to 2.0) | 1.10 (1.01-1.20) | 0.03 |
| All-cause death | 954 (15.1) | 1,171 (15.1) | 0.0 (-0.7 to 0.7) | 1.00 (0.92-1.09) | 0.92 |
| Cardiovascular death | 661 (10.5) | 823 (10.3) | -0.2 (-1.0 to 0.6) | 1.02 (0.92-1.13) | 0.69 |
| Myocardial infarction | 386 (6.1) | 521 (6.7) | 0.6 (-0.2 to 1.4) | 1.10 (0.97-1.26) | 0.14 |
| Hospitalization for heart failure | 198 (3.1) | 291 (3.7) | 0.6 (0.0 to 1.2) | 1.21 (1.01-1.45) | 0.04 |
| **DAPT duration ≥ 12 months** | **(N=26,319)** | **(N=38,000)** |  |  |  |
| Major adverse cardiovascular event* | 2,105 (8.0) | 3,408 (9.0) | 1.0 (0.5 to 1.4) | 1.13 (1.07-1.19) | <0.0001 |
| All-cause death | 1,733 (6.6) | 2,551 (6.7) | 0.1 (-0.3 to 0.5) | 1.02 (0.96-1.08) | 0.53 |
| Cardiovascular death | 1,273 (4.8) | 1,873 (4.9) | 0.1 (-0.3 to 0.5) | 1.02 (0.95-1.10) | 0.58 |
| Myocardial infarction | 803 (3.1) | 1,196 (3.1) | 0.1 (-0.2 to 0.4) | 1.03 (0.95-1.13) | 0.47 |
| Hospitalization for heart failure | 820 (3.1) | 1,589(4.2) | 1.0 (0.7 to 1.3) | 1.35 (1.24-1.47) | <0.0001 |

*Composite of cardiovascular death, myocardial infarction, and hospitalization for heart failure

DAPT, dual antiplatelet therapy; CI, confidence interval.

**Supplemental Table 7. Risks of primary and secondary outcomes at 5 years after percutaneous coronary intervention between patients prescribed with conventional or third-generation β-blockers.**

|  | **Conventional**  **β-blocker**  **(N=28,723)** | **Third-generation**  **β-blocker**^†^  **(N=17,023)** | **Risk difference**  **(95% CI)** | **Hazard ratio**  **(95% CI)** | **p*-*value** |
| --- | --- | --- | --- | --- | --- |
| Major adverse cardiovascular event* | 2,821 (9.8) | 1,733 (10.2) | 0.4 (-0.2 to 1.0) | 1.04 (0.98-1.10) | 0.24 |
| All-cause death | 2,377 (8.2) | 1,345 (7.9) | -0.3 (-0.9 to 0.3) | 0.95 (0.89-1.02) | 0.18 |
| Cardiovascular death | 1,716 (6.0) | 981 (5.8) | -0.2 (-0.7 to 0.2) | 0.96 (0.89-1.03) | 0.37 |
| Myocardial infarction | 1,087 (3.8) | 630 (3.7) | -0.1 (-0.5 to 0.3) | 0.98 (0.88 to 1.08) | 0.66 |
| Hospitalization for heart failure | 1,164 (4.1) | 715 (4.2) | 0.1 (-0.3 to 0.5) | 1.03 (0.94 to 1.11) | 0.45 |

* Composite of cardiovascular death, myocardial infarction, and hospitalization for heart failure

^†^ Third-generation β-blocker indicates carvedilol and nebivolol

**Supplemental Figure 1. Standardized mean difference before and after stabilized inverse probability of treatment weighting.**

**
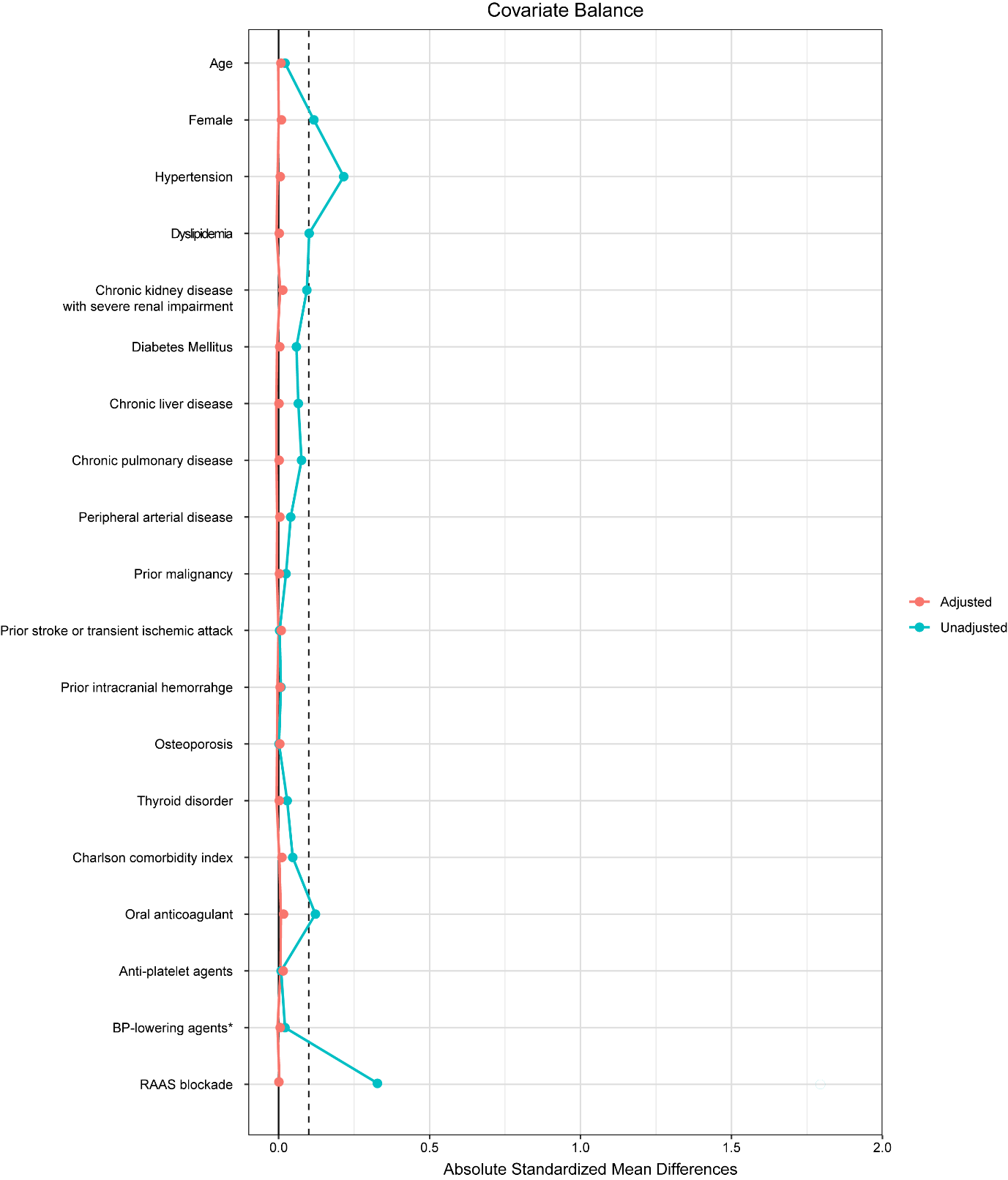
**

The standardized mean difference for each covariate before (green) and after (red) stabilized inverse probability of treatment weighting are presented. Standardized mean difference lesser than 0.10 was considered to be balanced. RAAS, renin-angiotensin-aldosterone system.

* Calcium channel blockers, α-adrenergic blockade, or diuretics

**Supplemental Figure 2. Distribution of stabilized inverse probability of treatment weightings**

**
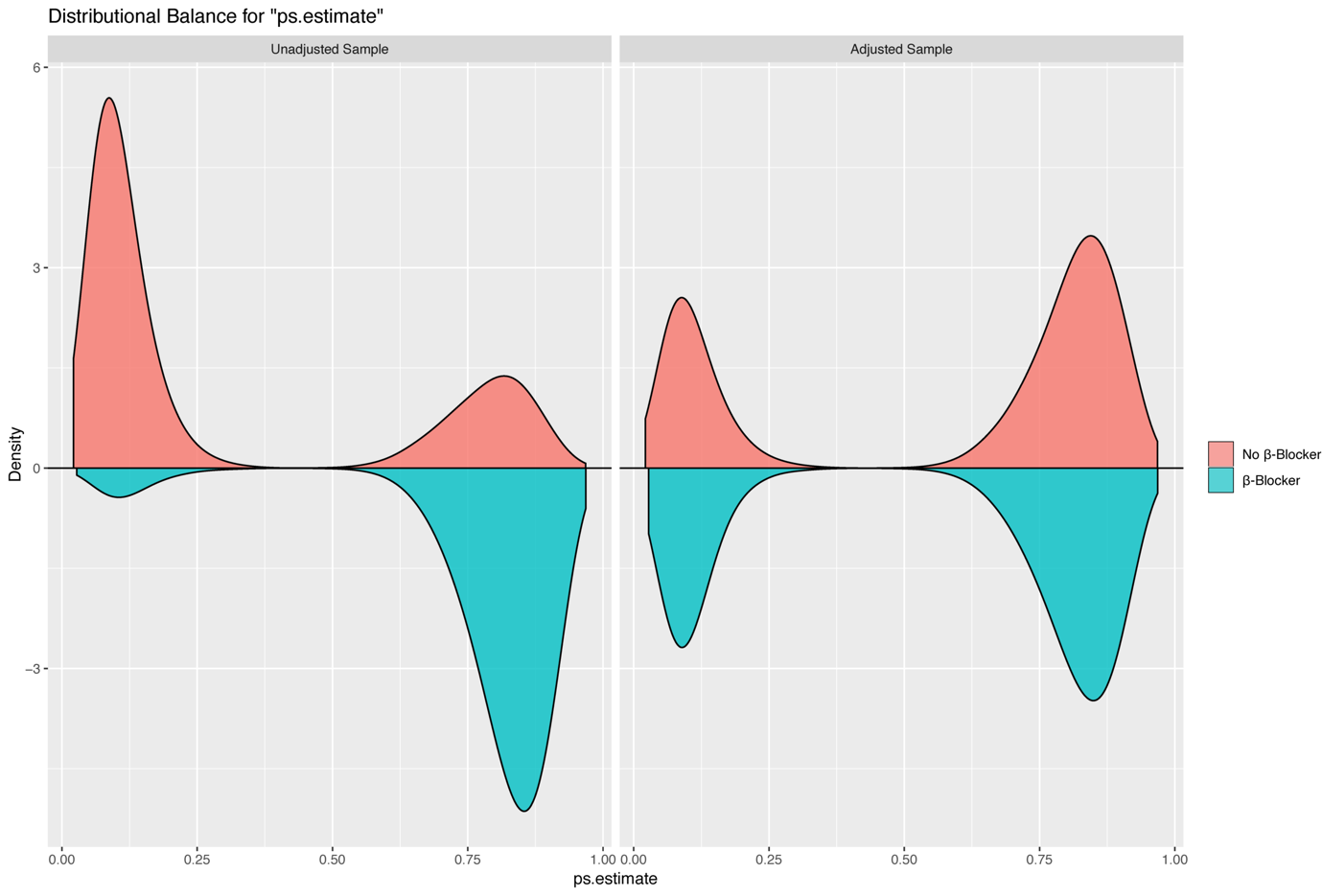
**

Density plot depicted as green color and red color indicates patients treated with and without β-blocker, respectively.

**Supplemental Figure 3. Time-to-event curves for all-cause death for 5 years after PCI**

**
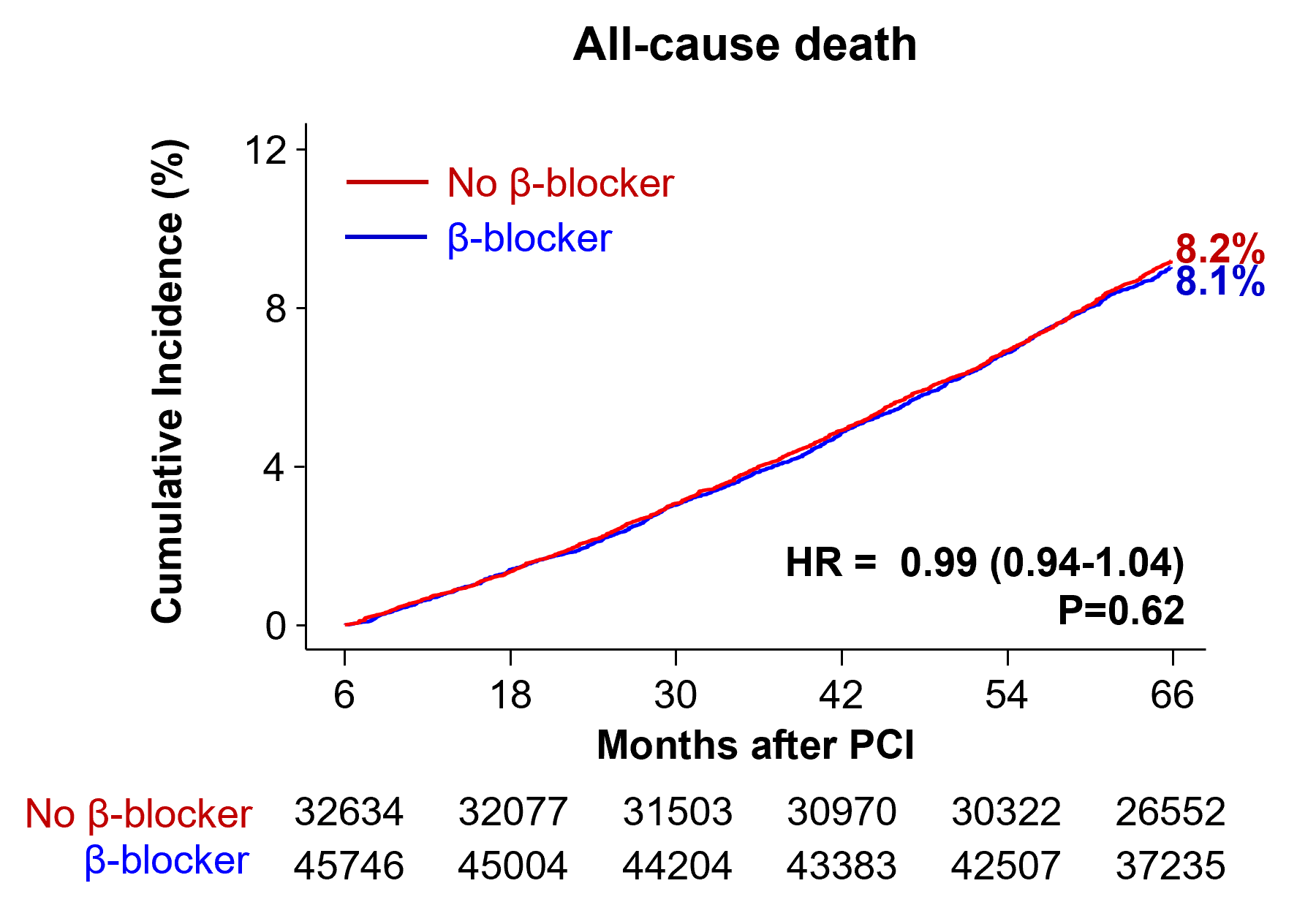
**

The cumulative incidence of all-cause death for 5 years after PCI. HR, hazard ratio; MI, myocardial infarction; PCI, percutaneous coronary intervention.

**Supplemental Figure 4. Subgroup analysis for all-cause death**

**
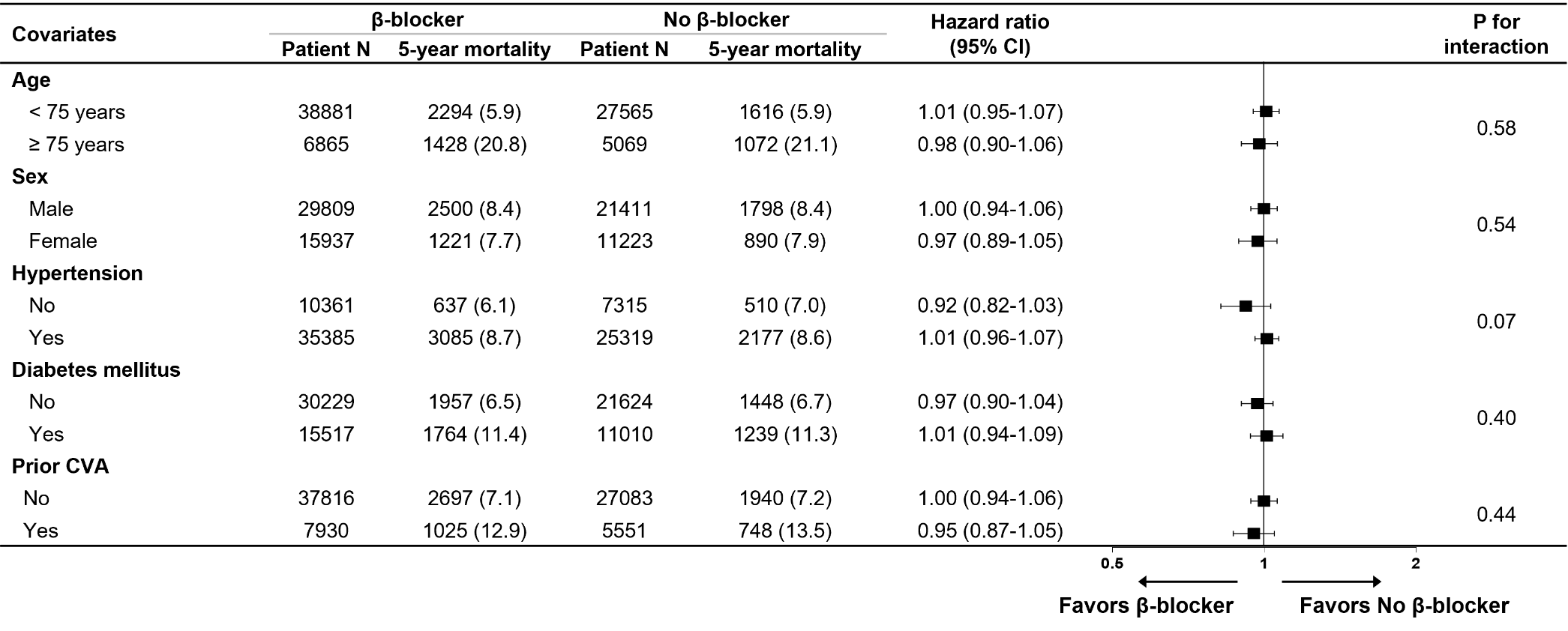
**

Numbers and percentages show the number of patients at risk and the all-cause mortality rate at 5 years after drug-eluting stent implantation, respectively. CI, confidence interval; CVA, cerebrovascular accidents.
